# Association Between Nucleated Red Blood Cell Counts and the Mortality in Patients With Liver Diseases: An Analysis of the MIMIC‐IV Database

**DOI:** 10.1111/jcmm.70982

**Published:** 2025-12-11

**Authors:** Yuanyuan Zhu, Danyang Yan, Fang Peng, Run Yao, Ning Li

**Affiliations:** ^1^ Department of Blood Transfusion, Clinical Transfusion Research Center, Xiangya Hospital Central South University Changsha Hunan Province China; ^2^ Department of Clinical Laboratory, Xiangya Hospital Central South University Changsha Hunan Province China; ^3^ National Clinical Research Center for Geriatric Disorders, Xiangya Hospital Central South University Changsha Hunan Province China; ^4^ Department of Clinical Laboratory Medicine, Xijing Hospital Fourth Military Medical University Xi'an Shanxi Province China; ^5^ NHC Key Laboratory of Cancer Proteomics, Xiangya Hospital Central South University Changsha Hunan Province China

**Keywords:** all‐cause mortality, liver diseases, MIMIC‐IV database, nucleated red blood cells

## Abstract

Liver disease is a leading cause of death worldwide. Nucleated red blood cells (NRBCs) are associated with high mortality and poor outcomes in patients with severe illnesses. However, the relationship between NRBCs and severe liver disease remains unclear. Potential confounding effects were managed using propensity score matching. The association between NRBCs and clinical outcomes in patients with liver disease was clarified using Cox proportional hazards regression analysis and smoothing splines. Differences in NRBCs between 30‐day survivors and non‐survivors within the pre‐matched cohort during the first 30 days after ICU admission were assessed using generalised additive mixed models. Compared to the 30‐day survivors, the 30‐day non‐survivors had significantly higher NRBC counts. Higher NRBC counts were significantly correlated with an augmented risk of 30‐day, 90‐day and in‐hospital mortality, with concurrently decreased hospitalisation durations. Inpatients with liver disease, progressive increases in the 30‐day mortality risk were associated with increased NRBC counts. The association between NRBCs and enhanced 30‐day mortality rates was consistent across stages and etiologies. Moreover, 30‐day non‐survivors experienced average daily increases in NRBC counts of 0.31% compared with 30‐day survivors. Elevated NRBC counts correlated with increased 30‐, 90‐day and in‐hospital mortality in patients with liver disease.

**Abbreviations**
AARC: APASL ACLF research consortium
Alb: albumin
ALT: alanine transaminase
AST: aspartate transaminase
BIDMC: Beth Israel Deaconess Medical Center
BUN: blood urea nitrogen
CECs: CD71^+^ erythroid cells
CI: confidence interval
COPD: chronic obstructive pulmonary disease
Cr: creatinine
GAMMs: Generalised Additive Mixed Models
HIV: human immunodeficiency virus
HR: hazard ratio
ICU: intensive care unit
INR: international normalised ratio
Lac: lactate
LOS hospital: length of hospital stay
LOS ICU: length of ICU stay
LRT: logarithmic likelihood ratio test
Meld: model for end‐stage liver disease
MIMIC‐IV: Medical Information Mart for Intensive Care‐IV
MIT: Massachusetts Institute of Technology
NRBCs: nucleated red blood cells
PLT: platelet count
PSM: propensity score matching
SMD: standardised mean difference
SOFA: Sequential Organ Failure Assessment
SQL: Structured Query Language
TBIL: total bilirubin
WBC: white blood cell count

## 1 Introduction

The liver, located in the upper right part of the abdomen, is vital for bilirubin and nutrient metabolism, blood volume regulation, bile secretion, detoxification, mineral and vitamin storage, synthesis of prothrombin, fibrinogen and clotting factors and immunoregulation [1]. Despite its robust functionality, various factors, such as drugs, environmental toxicants, viruses and dietary components, can impair its function [2]. When liver damage occurs, lipid metabolism in hepatocytes is dysregulated, liver function is impaired and the immune system is activated, leading to various liver diseases [3, 4, 5]. Liver diseases include viral hepatitis, nonalcoholic fatty liver disease, alcoholic liver disease, cirrhosis, liver failure, autoimmune liver disease and hepatocellular carcinoma (HCC) [6]. Each of these conditions poses a unique challenge to liver health, potentially leading to progressive dysfunction and posing significant risks to overall well‐being [7]. Worldwide, liver diseases have become a prominent factor in illness and death rates [8], leading to an annual loss of two million lives, representing 4% of global deaths [9]. Therefore, elucidating the mechanisms underlying liver disease development and identifying potential early biomarkers is essential.

Nucleated red blood cells (NRBCs) are progenitor cells involved in mammalian erythropoiesis [10]. In healthy adult humans, NRBCs are not found in peripheral blood, as nuclear extrusion occurs prior to their release into the circulation [11]. However, under certain pathological conditions, particularly those associated with severe anaemia or stress, the bone marrow may accelerate erythropoiesis and prematurely discharge NRBCs into peripheral blood [12]. Hypoxemia of the arterial system may contribute significantly to the formation of NRBCs [13]. The process by which NRBCs enter the bloodstream from the bone marrow is unclear, but their appearance in adult peripheral blood is generally considered an abnormal finding and may indicate underlying haematological disorders, malignancies or acute hemolytic processes, which are also signs of disease severity and an increase in mortality [14, 15, 16]. For example, the presence of NRBCs has been proposed as an indicator of overall in‐hospital mortality in individuals with sepsis, septic shock and cardiovascular diseases [17, 18, 19]. In addition, Li et al. observed CD71^+^ erythroid cells (including NRBCs and reticulocytes) among extramedullary haematopoietic cells in the liver at the time of biliary atresia diagnosis. They also discovered that preemptive depletion of hepatic CD71^+^ erythroid cells with antibodies safeguarded neonatal mice from rhesus rotavirus‐induced biliary atresia [20]. Jie et al. reported a significant increase in the number of CD45^+^CD71^+^ erythroid cells in HCC tissues. These cells exert immunosuppressive effects on the tumour microenvironment and can act as important biomarkers for predicting HCC recurrence [21].

However, a systematic and comprehensive assessment of the potential of NRBCs as predictors of liver disease remains lacking. Liver diseases might be of particular importance in this regard because the liver is an extramedullary haematopoietic organ. Hence, in this study, we analysed data on patients with liver disease from the Medical Information Market for Intensive Care IV (MIMIC‐IV) (v2.0) database, focusing on the association between NRBCs and prognosis in patients with liver disease. This study may provide insights into the predictive value of NRBCs for clinical outcomes in patients with liver disease, as well as new approaches toward improving prognoses in this population.

## 2 Materials and Methods

### 2.1 Study Design

This retrospective cohort study used data from the MIMIC‐IV database, which contained information on various clinical parameters. Permission to access the MIMIC‐IV database was obtained (Record ID: 40060500). To safeguard patient confidentiality, all personally identifiable information was de‐identified, and the requirement for informed consent was waived. The study was approved by the institutional review boards of the Massachusetts Institute of Technology (MIT) and the Beth Israel Deaconess Medical Center (BIDMC).

### 2.2 Patient Selection

The inclusion criteria were as follows: (1) liver disease diagnoses, including liver failure, cirrhosis, hepatitis (alcoholic, autoimmune and viral), hepatorenal syndrome, liver necrosis, liver tumour, fatty liver, hepatic coma, liver abscess and liver fibrosis; and (2) available NRBC data. The exclusion criteria were as follows: (1) haematological malignancies and other haematological diseases, (2) solid carcinomas, (3) immunosuppression and (4) treatment with chemotherapy and corticosteroids.

### 2.3 Data Collection

Structured Query Language (SQL) and PostgreSQL were used to extract data. The demographic data included age, sex, ethnicity and body mass index (BMI). The disease severity was assessed using the model for end‐stage liver disease (MELD) score [22], Sequential Organ Failure Assessment (SOFA) score [23] and APASL ACLF research consortium (AARC) score [24] for each patient. Baseline laboratory values included the platelet count (PLT); levels of alanine transaminase (ALT), aspartate transaminase (AST), albumin (Alb), total bilirubin (TBIL) and blood urea nitrogen (BUN); international normalised ratio (INR), lactate (Lac) levels; white blood cell count (WBC); and creatinine (Cr) level, based on the highest values recorded. Comorbid conditions included hypertension, diabetes, congestive heart failure, cerebrovascular disease, chronic obstructive pulmonary disease (COPD), renal disease and sepsis. Baseline NRBC counts were measured within the first 24 h of ICU admission, with the highest value selected in cases of multiple measurements. Subsequent NRBC counts were performed at irregular intervals.

The outcome measures included 30‐day mortality, 90‐day mortality, in‐hospital mortality, length of hospital stay (LOS‐hospital) and length of ICU stay (LOS‐ICU).

### 2.4 Statistical Analysis

Continuous variables are expressed as mean ± standard deviation or median with interquartile range and were analysed using the Student's *t*‐test for a normal distribution or Kruskal–Wallis *H* test for a non‐normal distribution. All patients were categorised based on 30‐day mortality outcomes. To determine the links between baseline variables and 30‐day mortality, univariate Cox analysis was performed.

Propensity score matching (PSM) was utilised to account for variations between the low NRBCs group (< 1.0%) and high NRBCs group (≥ 1.0%) [25]. Propensity scores were derived from demographic information, scoring systems, laboratory data and comorbidities. One‐to‐one nearest neighbour matching was applied, with a calliper width of 0.02. The patient outcomes in the two groups were analysed in both the pre‐ and post‐matched cohorts.

To determine the independent effects of the baseline NRBC count on 30‐day mortality, we applied multivariate Cox regression analysis and smooth curve fitting using both unadjusted and adjusted models. Moreover, a segmented regression model and logarithmic likelihood ratio test (LRT) were used to examine the threshold effect between NRBC counts and 30‐day mortality [26]. The risk of 30‐day death in patients with liver disease was stratified by disease process (e.g., hepatitis, cirrhosis or liver failure) and aetiology (e.g., viral infection or alcohol), and the interaction of NRBCs with these stratified groups was assessed.

Generalised Additive Mixed Models (GAMMs) were used to visualise and quantify the differences in NRBC count changes between 30‐day survivors and non‐survivors within the pre‐matched cohort during the first 30 days after ICU admission. GAMMs were well suited for analysing longitudinal data and effectively handled unbalanced and unequally spaced observations.

Statistical analyses were performed using R and EmpowerStats software. Statistical significance was set at *p* < 0.05.

## 3 Results

### 3.1 Participants Selection

**FIGURE 1:**
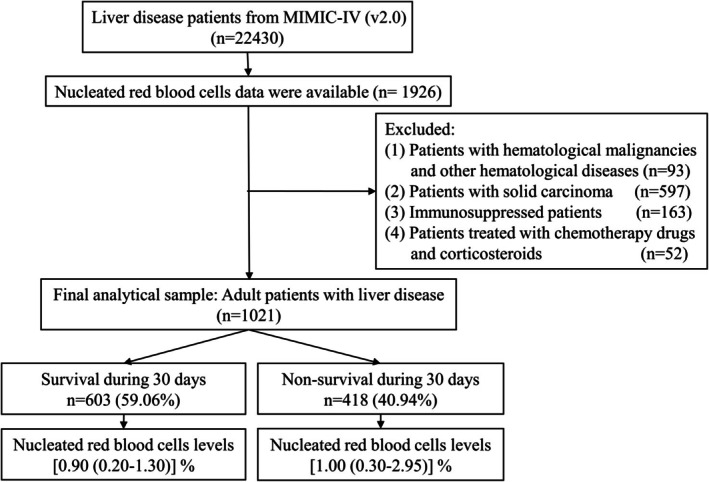
Flow chart of the study.

Overall, 22,430 adult patients with liver disease admitted to the ICU and with data in the MIMIC‐IV database (v2.0) were initially included. Of these, information on NRBCs was available for 1926 patients. We excluded patients with haematological malignancies and other haematological diseases (*n* = 93), solid carcinomas (*n* = 597) and immunosuppression (*n* = 163) and patients treated with chemotherapy drugs and corticosteroids (*n* = 52). Finally, 1021 patients were eligible: 603 participants in the 30‐day survivor group and 418 participants in the 30‐day non‐survivor group (Figure 1).

### 3.2 Baseline Patient Characteristics

Table 1 shows the baseline characteristics of the eligible patients with liver disease. Among them, 418 (40.94%) died within 30 days. Compared with the 30‐day survival group, the 30‐day non‐survivor group had higher NRBC counts [1.00 (0.30–2.95) vs. 0.90 (0.20–1.30)], ages [61.89 ± 15.16 vs. 56.40 ± 15.34], MELD scores [28.64 ± 9.12 vs. 23.24 ± 9.79], SOFA scores [12.00 (9.00–15.00) vs. 0.90 (0.20–1.30)], AARC scores [10.69 ± 1.58 vs. 9.86 ± 1.65], AST levels [200.00 (78.00–1073.75) vs. 169.00 (59.00–557.50)], TBIL levels [2.80 (1.40–9.88) vs. 2.60 (1.00–5.75)], BUN levels [42.00 (27.00–67.75) vs. 30.00 (18.00–54.00)], INR[2.30 (1.70–3.40) vs. 1.80 (1.40–2.50)], Lac levels [7.19 ± 4.75 vs. 5.32 ± 2.97], WBC counts [16.35 (10.60–23.78) vs. 13.90 (9.30–20.20)], Cr levels [2.30 (1.42–3.60) vs. 1.60 (1.00–3.10)], and sepsis rates [367 (87.80%) vs. 459 (76.12%)] and lower PLT counts [90.00 (55.00–152.00) vs. 114.00 (66.00–179.00)] and Alb levels [2.70 ± 0.67 vs. 2.84 ± 0.56].

**TABLE 1:** Baseline characteristics of total cohort, 30‐day survivors and 30‐day non‐survivors.

Variables	Total (*n* = 1021)	30‐day survivor (*n* = 603)	30‐day non survivor (*n* = 418)	*p*
Demographic data				
Age (year)	58.65 ± 15.49	56.40 ± 15.34	61.89 ± 15.16	< 0.001*
Gender				
Male	579 (56.71%)	346 (57.38%)	233 (55.74%)	0.603
Female	442 (43.29%)	257 (42.62%)	185 (44.26%)	
Ethnicity				
White	607 (59.45%)	376 (62.35%)	231 (55.26%)	< 0.001*
Black	121 (11.85%)	82 (13.60%)	39 (9.33%)	
Other	293 (28.70%)	145 (24.05%)	148 (35.41%)	
BMI (kg/m^2^)	30.49 ± 6.60	30.50 ± 6.00	30.49 ± 7.38	0.994
Score system				
Meld score	25.45 ± 9.88	23.24 ± 9.79	28.64 ± 9.12	< 0.001*
SOFA score	10.00 (6.00–13.00)	8.00 (6.00–11.00)	12.00 (9.00–15.00)	< 0.001*
AARC score	10.20 ± 1.67	9.86 ± 1.65	10.69 ± 1.58	< 0.001*
Laboratory indicators				
PLT (K/μL)	102.00 (59.00–169.00)	114.00 (66.00–179.00)	90.00 (55.00–152.00)	< 0.001*
ALT (IU/L)	80.00 (30.00–403.00)	80.00 (30.00–314.00)	83.50 (32.00–502.75)	0.802
AST (IU/L)	169.00 (68.00–759.00)	169.00 (59.00–557.50)	200.00 (78.00–1073.75)	< 0.001*
Alb (g/dL)	2.78 ± 0.61	2.84 ± 0.56	2.70 ± 0.67	< 0.001*
TBIL (mg/dL)	2.60 (1.10–7.30)	2.60 (1.00–5.75)	2.80 (1.40–9.88)	< 0.001*
BUN (mg/dL)	35.50 (20.00–59.00)	30.00 (18.00–54.00)	42.00 (27.00–67.75)	< 0.001*
INR	1.90 (1.50–2.90)	1.80 (1.40–2.50)	2.30 (1.70–3.40)	< 0.001*
Lac (mmol/L)	6.09 ± 3.91	5.32 ± 2.97	7.19 ± 4.75	< 0.001*
WBC (K/μL)	14.70 (9.90–21.80)	13.90 (9.30–20.20)	16.35 (10.60–23.78)	< 0.001*
Cr (mg/dL)	1.90 (1.10–3.40)	1.60 (1.00–3.10)	2.30 (1.42–3.60)	0.005*
Comorbidity				
Hypertension	571 (55.93%)	346 (57.38%)	225 (53.83%)	0.261
Diabetes	304 (29.77%)	180 (29.85%)	124 (29.67%)	0.949
Congestive heart failure	346 (33.89%)	198 (32.84%)	148 (35.41%)	0.393
Cerebrovascular disease	108 (10.58%)	60 (9.95%)	48 (11.48%)	0.434
COPD	248 (24.29%)	139 (23.05%)	109 (26.08%)	0.268
Renal disease	259 (25.37%)	152 (25.21%)	107 (25.60%)	0.888
Sepsis	826 (80.90%)	459 (76.12%)	367 (87.80%)	< 0.001*
NRBCs (%)	1.00 (0.30–2.00)	0.90 (0.20–1.30)	1.00 (0.30–2.95)	< 0.001*

*Note:* * indicates statistical significance.

### 3.3 Baseline Demographic and Clinical Characteristics in the Low and High NRBC Groups

The 1021 eligible patients were divided into a high NRBC group (≥ 1.0%) and a low NRBC group (< 1.0%) based on NRBC counts. The baseline demographic and clinical characteristics of the patients with liver disease in the two groups are presented in Table 2. Before matching, the two groups showed significant differences in sex; SOFA scores; ALT, AST, Alb and Lac levels; and rates of diabetes, congestive heart failure and sepsis (*p* < 0.05). After matching, no significant between‐group differences were observed (*p* > 0.05).

**TABLE 2:** The baseline demographic and clinical characteristics of patients with liver disease in the high and low NRBC groups before and after PSM.

Variable	Before match				After match			
	Low NRBC group (*n* = 462) (< 1.0%)	High NRBC group (*n* = 559) (≥ 1.0%)	*p*	SMD	Low NRBC group (*n* = 433) (< 1.0%)	High NRBC group (*n* = 433) (≥ 1.0%)	*p*	SMD
Demographic data								
Age (year)	59.02 (14.90)	58.34 (15.97)	0.486	0.04	58.84 (14.93)	58.67 (15.77)	0.878	0.01
Gender								
Male	278 (60.17%)	301 (53.85%)	0.042	0.13	252 (58.20%)	236 (54.50%)	0.273	0.07
Female	184 (39.83%)	258 (46.15%)			181 (41.80%)	197 (45.50%)		
Ethnicity								
White	281 (60.82%)	326 (58.32%)	0.719	0.05	262 (60.51%)	244 (56.35%)	0.351	0.10
Black	53 (11.47%)	68 (12.16%)			49 (11.32%)	61 (14.09%)		
Other	128 (27.71%)	165 (29.52%)			122 (28.18%)	128 (29.56%)		
BMI (kg/m^2^)	30.75 (6.73)	30.29 (6.48)	0.266	0.07	30.71 (6.88)	30.54 (6.58)	0.713	0.03
Score system								
Meld score	25.11 (9.98)	25.73 (9.80)	0.322	0.06	25.18 (9.81)	25.64 (10.05)	0.498	0.05
SOFA score	9.00 (6.00–12.00)	10.00 (7.00–14.00)	< 0.001	0.24	9.00 (6.00–13.00)	10.00 (6.00–13.00)	0.249	0.08
AARC score	10.17 (1.65)	10.22 (1.69)	0.641	0.03	10.19 (1.65)	10.24 (1.71)	0.701	0.03
Laboratory data								
PLT (K/μL)	107.00 (65.00–171.00)	99.00 (56.50–164.50)	0.236	0.07	105.00 (64.00–170.00)	102.00 (60.00–165.00)	0.406	0.06
ALT (IU/L)	80.00 (27.00–217.00)	80.00 (37.00–574.00)	< 0.001	0.11	80.00 (28.00–232.00)	80.00 (36.00–503.00)	0.229	0.08
AST (IU/L)	143.00 (54.25–434.25)	201.00 (84.50–1220.00)	< 0.001	0.13	148.00 (58.00–474.00)	172.00 (77.00–1044.00)	0.287	0.07
Alb (g/dL)	2.87 (0.59)	2.71 (0.62)	< 0.001	0.27	2.84 (0.58)	2.80 (0.61)	0.353	0.06
TBIL (mg/dL)	2.60 (1.10–6.97)	2.60 (1.20–7.45)	0.949	0.00	2.60 (1.10–7.30)	2.60 (1.20–7.30)	0.867	0.01
BUN (mg/dL)	34.00 (19.25–60.00)	36.00 (21.50–57.50)	0.923	0.01	34.00 (19.00–60.00)	36.00 (21.00–58.00)	0.940	0.01
INR	1.95 (1.50–2.80)	1.90 (1.50–2.95)	0.992	0.00	2.00 (1.50–2.80)	1.90 (1.50–3.00)	0.670	0.03
Lac (mmol/L)	5.76 (3.34)	6.36 (4.30)	0.015	0.15	5.81 (3.42)	6.00 (3.95)	0.441	0.05
WBC (K/μL)	15.45 (10.30–21.08)	14.40 (9.55–22.65)	0.873	0.01	15.60 (10.30–21.40)	14.20 (9.20–22.70)	0.594	0.04
Cr (mg/dL)	1.80 (1.10–3.30)	2.10 (1.20–3.40)	0.804	0.02	1.80 (1.10–3.30)	2.10 (1.10–3.50)	0.868	0.01
Comorbidities								
Hypertension	268 (58.01%)	303 (54.20%)	0.223	0.08	247 (57.04%)	247 (57.04%)	1.000	0.00
Diabetes	155 (33.55%)	149 (26.65%)	0.016	0.15	137 (31.64%)	125 (28.87%)	0.375	0.06
Congestive heart failure	172 (37.23%)	174 (31.13%)	0.040	0.13	156 (36.03%)	140 (32.33%)	0.252	0.08
Cerebrovascular disease	58 (12.55%)	50 (8.94%)	0.062	0.12	47 (10.85%)	43 (9.93%)	0.656	0.03
COPD	111 (24.03%)	137 (24.51%)	0.858	0.01	107 (24.71%)	105 (24.25%)	0.874	0.01
Renal disease	129 (27.92%)	130 (23.26%)	0.088	0.11	117 (27.02%)	112 (25.87%)	0.700	0.03
Sepsis	356 (77.06%)	470 (84.08%)	0.004	0.18	341 (78.75%)	348 (80.37%)	0.555	0.04

### 3.4 Outcomes of Patients With Liver Disease in NRBC Groups

In the pre‐matched cohort, the 30‐day [46.51% vs. 34.20%], 90‐day [55.28% vs. 43.94%] and in‐hospital mortality [48.12% vs. 35.28%] rates were higher in the high NRBC group than in the low NRBC group. Compared to the low NRBC group, the risk of 30‐day, 90‐day and in‐hospital mortality increased by 51% [hazard ratio (HR): 1.51, 95% confidence interval (CI): 1.24–1.84], 43% (HR: 1.43, 95% CI: 1.19–1.70) and 70% (HR: 1.70, 95% CI: 1.32–2.19), respectively, in the high NRBC group. The LOS‐hospital were 16.71 (8.73–27.12) and 4.63 (2.31–10.76) days, respectively, in the low NRBC group and 14.73 (7.84–25.18) and 5.71 (2.04–12.47) days, respectively, in the high NRBC group.

Similar results were observed in the post‐matching cohort. The 30‐day [45.03% vs. 35.33%], 90‐day [54.50% vs. 44.11%] and in‐hospital [46.19% vs. 36.26%] mortality rates were significantly higher in the high NRBC group than in the low NRBC group. Compared to the low NRBC group, the risk of 30‐day, 90‐day and in‐hospital mortality increased by 37% [HR: 1.37, 95% CI: 1.11–1.70], 36% (HR: 1.36, 95% CI: 1.12–1.64) and 51% (HR: 1.51, 95% CI: 1.15–1.98), respectively, in the high NRBC group. The LOS‐hospital was longer in the low NRBC group [16.36 (8.41–27.23)] than in the high NRBC group [14.51 (8.06–24.75)] (Table 3).

**TABLE 3:** Outcomes of the patients with liver disease in the low and high NRBC groups.

Variables	Low NRBC group	High NRBC group	HR (95% CI)	*p*
	(< 1.0%)	(≥ 1.0%)		
**Pre‐matched cohort**	***N* = 462**	***N* = 559**		
30‐day mortality	158 (34.20%)	260 (46.51%)	1.51 (1.24, 1.84)	< 0.001*
90‐day mortality	203 (43.94%)	309 (55.28%)	1.43 (1.19, 1.70)	< 0.001*
In‐hospital mortality	163 (35.28%)	269 (48.12%)	1.70 (1.32, 2.19)	< 0.001*
LOS hospital	16.71 (8.73–27.12)	14.73 (7.84–25.18)	−2.63 (−5.18, −0.07)	0.021*
LOS ICU	4.63 (2.31–10.76)	5.71 (2.04–12.47)	1.44 (0.36, 2.52)	0.131
**Post‐matched cohort**	***N* = 433**	***N* = 433**		
30‐day mortality	153 (35.33%)	195 (45.03%)	1.37 (1.11, 1.70)	0.004*
90‐day mortality	191 (44.11%)	236 (54.50%)	1.36 (1.12, 1.64)	0.002*
In‐hospital mortality	157 (36.26%)	200 (46.19%)	1.51 (1.15, 1.98)	0.003*
LOS hospital	16.36 (8.41–27.23)	14.51 (8.06–24.75)	−2.81 (−5.53, −0.09)	0.031*
LOS ICU	4.61 (2.32–10.76)	5.24 (1.94–10.97)	0.78 (−0.34, 1.90)	0.643

*Note:* * indicates statistical significance.

### 3.5 Association Between NRBC Counts and 30‐Day Mortality in the Pre‐Matched Cohort of Patients With Liver Disease

**FIGURE 2:**
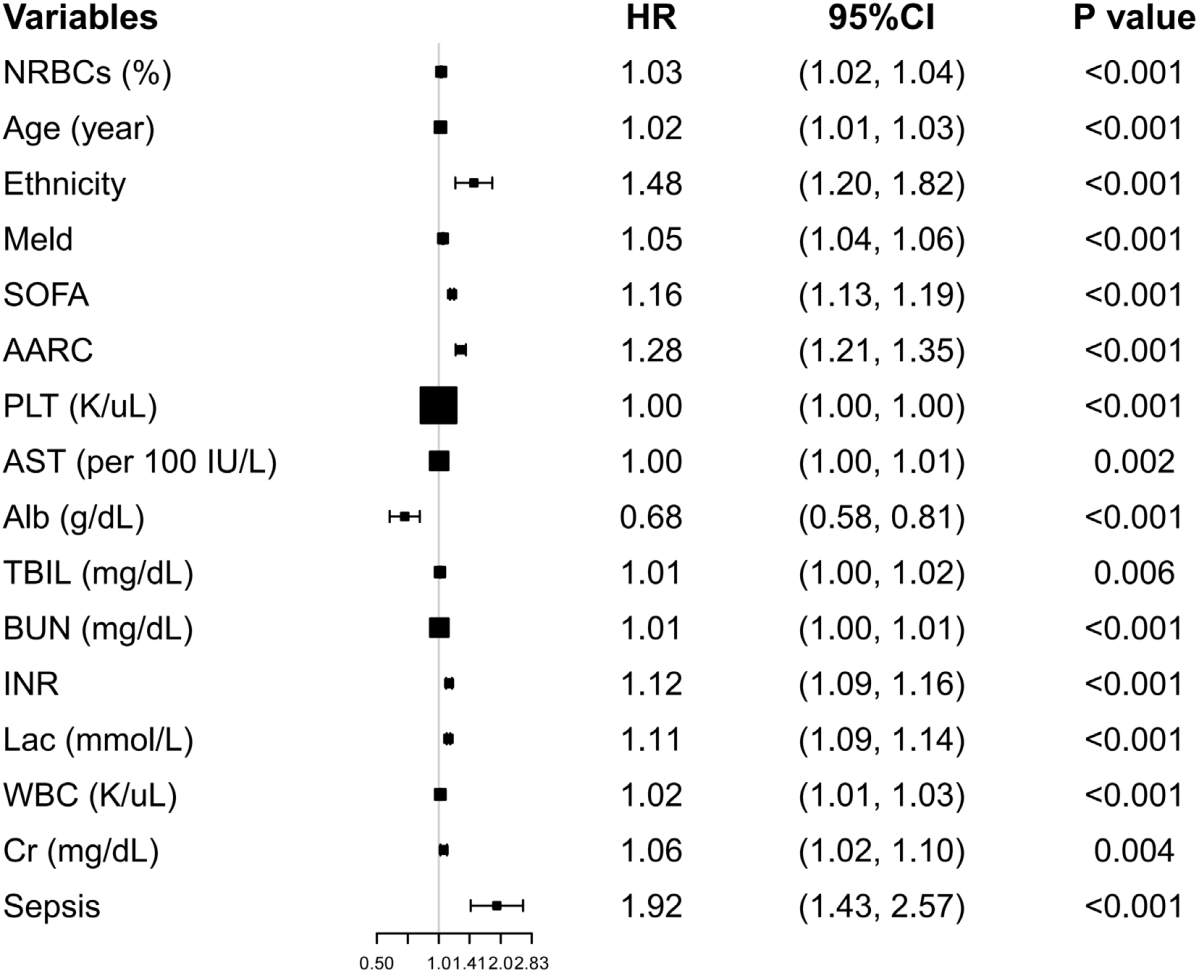
Variables related to 30‐day mortality.

Univariate Cox analysis showed that NRBC counts were positively associated with the 30‐day mortality (HR: 1.03, 95% CI: 1.02–1.04). In addition, age (HR: 1.02, 95% CI: 1.01–1.03), ethnicity (HR: 1.48, 95% CI: 1.20–1.82), MELD score (HR: 1.05, 95% CI: 1.04–1.06), SOFA score (HR: 1.16, 95% CI: 1.13–1.19), AARC score (HR: 1.28, 95% CI: 1.21–1.35), AST level (HR: 1.00, 95% CI: 1.00–1.01), TBIL level (HR: 1.01, 95% CI: 1.00–1.02), BUN level (HR: 1.01, 95% CI: 1.00–1.01), INR (HR: 1.12, 95% CI: 1.09–1.16), Lac level (HR: 1.11, 95% CI: 1.09–1.14), WBC count (HR: 1.02, 95% CI: 1.01–1.03), Cr level (HR: 1.06, 95% CI: 1.02–1.10) and sepsis rate (HR: 1.92, 95% CI: 1.43–2.57) were positively correlated with 30‐day mortality, while PLT counts (HR: 1.00, 95% CI: 1.00–1.00) and Alb levels (HR: 0.68, 95% CI: 0.58–0.81) were negatively correlated (Figure 2).

The results of the multivariate Cox regression analysis are presented in Table 4. A positive association was found between the NRBC count as a continuous variable and 30‐day mortality in the unadjusted model (HR: 1.03, 95% CI: 1.02–1.04), adjusted model I (HR: 1.03, 95% CI: 1.02–1.04) and adjusted model II (HR: 1.02, 95% CI: 1.01–1.03). When the NRBC count was used as a dichotomous categorical variable, compared to the low group (< 1.0%), the risk of 30‐day mortality in the high group (≥ 1.0%) increased by 51%, 51% and 29% in the unadjusted model, model I and model II, respectively. When the NRBC count was categorised into quartiles, the Q4 group (≥ 2.0%) had the highest mortality risk in the unadjusted model (HR: 1.68, 95% CI: 1.29–2.19), compared to the Q1 group (≤ 0.2%). Similar results were observed in adjusted model I (HR: 1.63, 95% CI: 1.25–2.12) and adjusted model II (HR: 1.35, 95% CI: 1.03–1.78). For the sensitivity analysis, the NRBC count quartile categorical variables were considered continuous variables, and the trend in the association between the NRBC count and 30‐day mortality was consistent (*p* < 0.05).

**TABLE 4:** Multivariable cox regression analysis of baseline NRBC for 30‐day mortality in the pre‐matched cohort.

Exposure	HR (95% CI)		
	Non‐adjusted model	Adjusted model I	Adjusted model II
NRBCs	1.03 (1.02, 1.04)*	1.03 (1.02, 1.04)*	1.02 (1.01, 1.03)*
NRBCs dichotomous			
Low (< 1.0%)	Ref	Ref	Ref
High (≥ 1.0%)	1.51 (1.24, 1.84)*	1.51 (1.24, 1.84)*	1.29 (1.05, 1.58)*
NRBCs quartile			
Q1 (≤ 0.2%)	Ref	Ref	Ref
Q2 (0.3%–0.9%)	0.87 (0.63, 1.19)	0.81 (0.59, 1.10)	0.88 (0.64, 1.21)
Q3 (1.0%–1.9%)	1.17 (0.89, 1.55)	1.14 (0.86, 1.50)	1.07 (0.80, 1.42)
Q4 (≥ 2.0%)	1.68 (1.29, 2.19)*	1.63 (1.25, 2.12)*	1.35 (1.03, 1.78)*
*p* value for trend	< 0.001	< 0.001	0.010

*Note:* Adjusted model I: adjust for age; gender; ethnicity; BMI. Adjusted model II: adjust for age; gender; ethnicity; BMI; Meld; SOFA; ARRC; PLT; AST; Alb; INR; Lac; WBC; TBIL; BUN; Cr; hypertension; sepsis. * indicates statistical significance.

### 3.6 Nonlinear Relationship Between NRBCs and 30‐Day Mortality Risk Among Patients With Liver Disease

**FIGURE 3:**
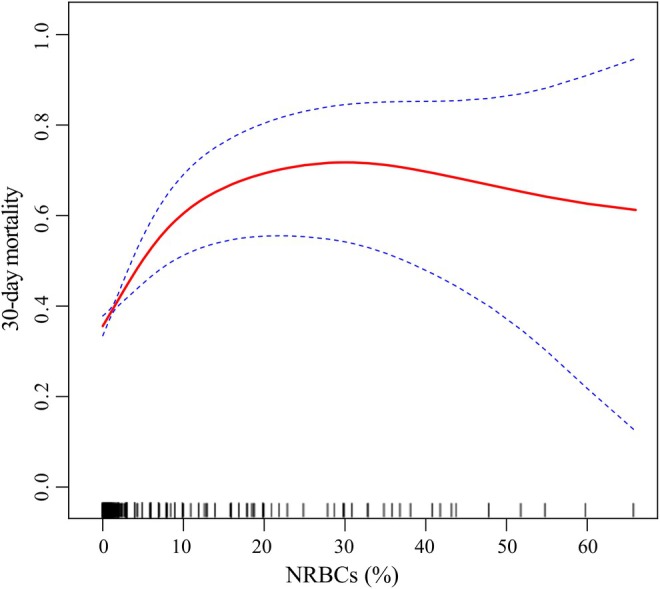
Smoothing spline fitting curve.

Smoothing splines were used todepict the relationship between NRBCs and 30‐day mortality in individuals with liver disease. Using a segmentation regression model, we identified the inflection point of the NRBC count as 7.0% (Table 5). After adjustment, when the NRBC count was < 7.0%, the mortality risk increased by 8.9% for each unit increment in NRBC count (HR: 1.09; *p* = 0.001). A *p*‐value of 0.010 for NRBCs indicated a nonlinear correlation between the NRBC count and 30‐day mortality risk in patients with liver disease (Table 5, Figure 3).

**TABLE 5:** Threshold effect analysis.

Models	Unadjusted		Adjusted	
	HR (95% CI)	*p*	HR (95% CI)	*p*
Model I				
One line slope	1.03 (1.02, 1.04)	< 0.001	1.02 (1.01, 1.03)	0.004
Model II				
Turning point (K1)				
< 7.0 slope 1	1.15 (1.10, 1.21)	< 0.001	1.09 (1.03, 1.15)	0.001
> 7.0 slope 2	1.00 (0.99, 1.02)	0.862	1.00 (0.98, 1.02)	0.991
LRT	< 0.001^a^		0.010^a^	

*Note:* Data are presented as HR (95% CI) and *p*‐value; LRT, Logarithmic likelihood ratio test (*p* < 0.05 means model II is significantly different from model I, which indicates a nonlinear association). The adjusted variables included age, gender, ethnicity, BMI, Meld, SOFA, ARRC, PLT, AST, Alb, INR, Lac, WBC, TBIL, BUN, Cr, hypertension and sepsis.

^a^ Indicates that model II is significant different from model I. Model I, linear analysis; model II, nonlinear analysis.

### 3.7 Subgroup Analyses of the Effect of NRBCs on 30‐Day Mortality in Patients With Liver Disease

We analysed the effect of NRBCs on 30‐day mortality rates in different liver diseases. NRBCs correlated with an increase in 30‐day mortality among patients with hepatitis (HR: 1.03, 95% CI: 1.02–1.05), cirrhosis (HR: 1.05, 95% CI: 1.02–1.07), other liver diseases (HR: 1.03, 95% CI: 1.02–1.05) and liver failure (HR: 0.99, 95% CI: 0.96–1.03) (Table 6). In addition, logistic regression analysis showed NRBCs were associated with increased mortality among patients with different etiologies, including viral infections (HR: 1.07, 95% CI: 1.04–1.10), alcoholism (HR: 1.03, 95% CI: 1.01–1.04) and other etiologies (HR: 1.02, 95% CI: 1.00–1.03). Additionally, interaction analyses revealed significant interaction terms between disease processes (*p* = 0.030) and etiologies (*p* = 0.027) and 30‐day mortality risk.

**TABLE 6:** Subgroup analyses of the effect of NRBCs on 30‐day mortality in patients with liver disease.

Subgroups	*N*	30‐day mortality		
		HR (95% CI)	*p*	*p* for interaction
Disease process				
Hepatitis	369	1.03 (1.02, 1.05)	< 0.001*	0.030*
Cirrhosis	232	1.05 (1.02, 1.07)	< 0.001*	
Liver failure	227	0.99 (0.96, 1.03)	0.697	
Other	193	1.03 (1.02, 1.05)	< 0.001*	
Aetiology				
Virus infection	205	1.07 (1.04, 1.10)	< 0.001*	0.027*
Alcoholic	273	1.03 (1.01, 1.04)	< 0.001*	
Other	543	1.02 (1.00, 1.03)	0.028*	

*Note:* * indicates statistical significance.

### 3.8 Correlation of NRBC Count Changes With 30‐Day Mortality in Patients With Liver Disease

**FIGURE 4:**
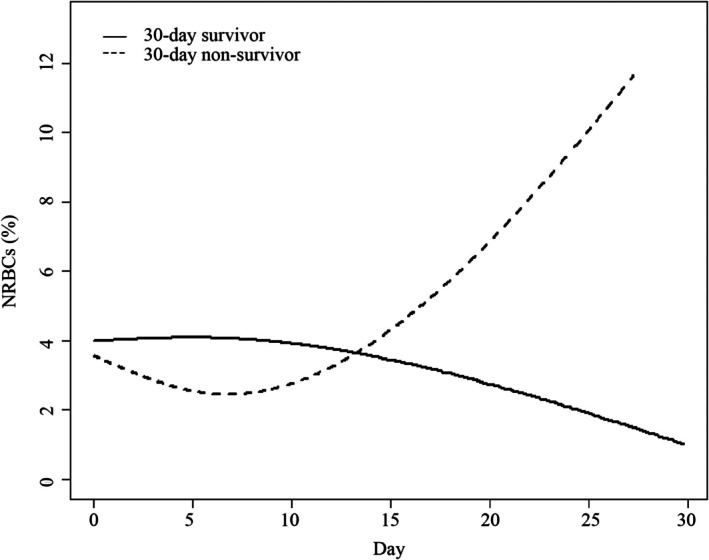
Association between changes in NRBCs and 30‐day mortality.

The changes in NRBC counts over time between the two groups are shown in Figure 4 and Table 7. In the 30‐day survivor group, NRBC counts decreased over time, whereas in the 30‐day non‐survivor group, NRBC counts first declined and then continued to increase (Figure 4). The 30‐day non‐survivors experienced an average increase of 0.31% per day in NRBC counts compared to the 30‐day survivors (Table 7).

**TABLE 7:** Relationship between changes (0–30 days) NRBC counts and 30‐day mortality in patients with liver disease derived from a generalised additive mixed model (GAMM).

Models	*β* (95% CI)	*p*
Day	−0.08 (−0.20, 0.04)	0.184
Death	0.89 (−1.11, 2.90)	0.384
Day × death	0.31 (0.12, 0.50)	0.002*

*Note:* Day indicated the mean of the increasing of indexes daily in 30‐day survival patients. Death indicated the difference of indexes at day 0 in 30‐day non‐survival patients compared with 30‐day survival patients. Day × Death indicated the average increasing of indexes daily in 30‐day non‐survival patients compared with 30‐day survival patients. * indicates statistical significance.

## 4 Discussion

NRBCs are typically absent from the peripheral blood of healthy adults. While previous studies have indicated that NRBCs in peripheral blood are linked to a poorer prognosis in various diseases, their clinical importance in liver diseases remains uncertain. This study explored the potential of NRBCs as predictors of mortality in a diverse group of patients with liver disease using the MIMIC‐IV (v2.0) database. First, we found that compared with the 30‐day survival group, the 30‐day mortality group had higher NRBC counts. All patients were categorised into low‐ and high‐NRBC groups based on their NRBC counts. High NRBC counts were linked to increasing 30‐day, 90‐day and in‐hospital mortalities but reduced LOS‐hospital. This may be due to the high mortality rate of patients in the high NRBC count group, resulting in a corresponding reduction in the average LOS‐hospital. Tayyab et al. [27] reported similar findings. The authors found that ICU patients with NRBCs had significantly higher mortality rates than those without NRBCs. Sarah et al. [28] observed that NRBCs were associated with notably higher mortality in neonatal ICU patients. These results reinforce the significant association between NRBCs and increased disease‐related mortality.

Next, after adjusting for different covariates, we analysed the relationship between NRBCs and mortality in different models. Multivariate Cox regression analysis revealed that the association between the NRBC count and mortality was consistent among the different models. In addition, the mortality risk increased with NRBC counts < 7.0 but not with NRBC counts > 7.0. Further subgroup analyses of the effect of NRBCs on 30‐day mortality in patients with different liver diseases indicated that NRBCs were associated with higher 30‐day mortality in patients with hepatitis, cirrhosis and other liver disease processes in addition to liver failure. Krieg et al. [29] reported similar findings inpatients receiving surgical intensive care. They found that the presence of NRBCs in the blood of patients in surgical intensive care has prognostic significance for patient mortality but is not linked to liver or kidney organ failure [29]. Lastly, from a GAMM, we found that 30‐day non‐survivors experienced an average increase in NRBC counts of 0.31% per day compared to 30‐day survivors, suggesting that NRBCs could be a useful tool for identifying critically ill patients with liver disease at a high risk of death. Furthermore, we found that patients with high NRBC counts had an average daily increase in the TBIL level (0.16 mg/dL), DBIL level (0.26 mg/dL), IBIL level (0.05 mg/dL), Cre level (0.01 mg/dL), INR (0.01) and WBC count (0.09 K/μL) compared with patients with low NRBC counts (Figure S1, Table S1), suggesting that NRBCs may affect the mortality of patients with liver disease by aggravating liver and kidney injuries.

However, the specific biological mechanisms linking NRBCs to the onset and progression of liver disease and mortality remain unclear. These potential pathways may be involved in immune regulation and responses. As the precursors of mature red blood cells, NRBCs have a vital but frequently neglected function in the immunological landscape of disorders [30, 31]. At their nucleation stage, NRBCs possess unique properties that intertwine with immune regulation and responses. Immunologically, NRBCs express both CD71 and CD235a in humans and CD71 and TER119 in mice, known as CD71^+^ erythroid cells (CECs), which include NRBCs and reticulocytes [32, 33]. CECs influence the host defence system in several ways. They have been implicated in modulating inflammatory responses through the secretion of cytokines and the expression of pattern recognition receptors, such as arginase‐2, TGF‐β, galectins, PD‐1: PDL‐1/PDL‐2, VISTA and ROS, which enable them to participate in pathogen detection and phagocytosis [31, 34, 35, 36]. Moreover, CECs can present antigens to T cells, thereby contributing to adaptive immunity [37]. The immunosuppressive and immunomodulatory properties of CECs have been observed in several diseases. In human immunodeficiency virus (HIV)‐infected patients, the CEC frequency is positively related to the plasma viral load. In addition, HIV particles capable of infection are located inside CECs but are absent in mature red blood cells. More significantly, CECs, through ROS, enhance HIV replication and infection in CD4^+^ T cells, making them more prone to HIV infection [38]. In patients with sepsis, CECs are expanded in the peripheral blood, and their frequencies are positively correlated with high levels of interleukin (IL)‐6 and interferon (IFN)‐γ. Furthermore, CECs may act as standalone indicators of the onset of hospital‐acquired infections and mortality within 30 days [39]. All these studies emphasise the crucial function of CECs in immunomodulation. Our findings indicate that NRBCs are crucial for the progression of liver diseases. On this basis, additional research is required to ascertain whether better management of NRBCs will enhance clinical outcomes, how NRBCs function in liver diseases (e.g., by modulating the immune response), and whether NRBCs could serve as a new target for improving prognoses and clinical outcomes in this population.

Research on liver disease biomarkers presents both promises and challenges in disease diagnosis and prognostic evaluation. The specificities and sensitivities of these biomarkers are critical. Currently, the diagnostic accuracy of many identified biomarkers requires further improvement because false‐positive or false‐negative results limit their clinical application. For example, alpha‐fetoprotein (AFP), a commonly used biomarker for HCC, may not be elevated in some patients with HCC and can yield false‐positive results in certain benign liver diseases, thereby compromising diagnostic reliability [40]. Additionally, variations in detection methods and standards across studies hinder the comparability and validation of results, further complicating the clinical application of biomarkers. Research on liver disease biomarkers offers numerous opportunities. With continuous technological advancements, the development of multiomics technologies, such as genomics, transcriptomics, proteomics and metabolomics, provides powerful tools for discovering additional potential biomarkers. By integrating and analysing biological information across different levels, it is possible to identify biomarker combinations with higher specificity and sensitivity, thereby improving the accuracy of disease diagnosis and prognosis evaluation. Furthermore, the rise of interdisciplinary research has fostered collaboration across fields such as medicine, biology and engineering, offering new perspectives and methodologies for biomarker research and applications. For instance, advances in nanotechnology and biosensors have enabled more sensitive and convenient detection methods for biomarkers. In light of these challenges and opportunities, collaborative efforts among researchers, clinicians and policymakers are essential to advance the study of liver disease biomarkers and improve patient health outcomes.

The major strength of this study was the evidence that elevated NRBC counts independently contribute to a higher mortality risk in patients with liver disease. In addition, dynamic changes in NRBC counts were available. Therefore, we assessed the predictive ability of changes in NRBC counts. However, this study has several limitations. First, residual confounding factors may have affected the clinical outcomes despite the use of multivariate adjustment and subgroup analyses; therefore, additional prospective case–control studies are needed. Secondly, this study did not explore the mechanism by which NRBCs affect liver diseases, and more experiments are needed to delve deeper into the mechanisms involved.

## 5 Conclusion

In summary, our results indicated that elevated NRBC counts are correlated with an increased risk of 30‐day, 90‐day and in‐hospital mortality in critically ill patients with liver diseases, confirming the significant link between NRBCs in the peripheral blood and higher mortality rates from diseases. Monitoring NRBC counts may aid in decision‐making and the management of liver diseases in clinical settings.

## Author Contributions

Y.Z.: Writing – original draft; D.Y.: Writing – review and editing; F.P.: Formal analysis; R.Y.: Software; N.L.: Funding acquisition. All authors read and approved the final manuscript.

## Funding

This work was funded by the National Natural Science Foundation of China (No. 82571796) and the Clinical Research Center Foundation of Xiangya Hospital (No. LN2021XYSX).

## Ethics Statement

The study employed public data sourced from the MIMIC‐IV database, with the data being de‐identified and not altered or combined in any way that would jeopardise participant confidentiality. Thus, the research does not need any more approval or extra consent.

## Consent

The authors have nothing to report.

## Conflicts of Interest

The authors declare no conflicts of interest.

## Supporting information


**Data S1:** jcmm70982‐sup‐0001‐DataS1.docx.

## Data Availability

The data that support the findings of this study are available from the corresponding author upon reasonable request.
