# Supplementary material for: Association Between Nucleated Red Blood Cell Counts and the Mortality in Patients With Liver Diseases: An Analysis of the MIMIC‐IV Database
Source: J Cell Mol Med. 2025 Dec 11;29(23):e70982. doi: 10.1111/jcmm.70982 (PMC12698331; doi:10.1111/jcmm.70982)
Supplement: Supplementary file 1 — Data S1: jcmm70982‐sup‐0001‐DataS1.docx. [file JCMM-29-e70982-s001.docx]

**
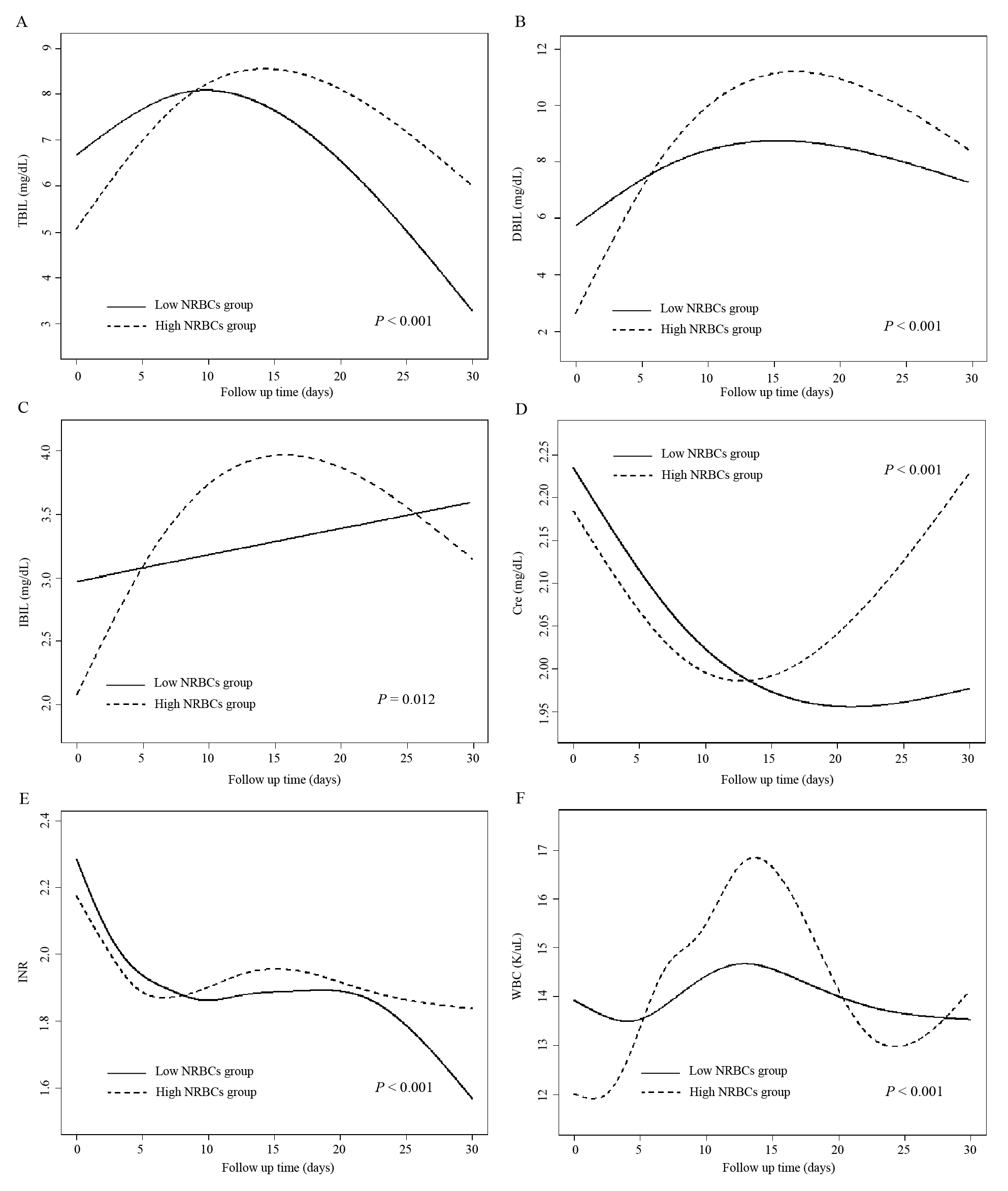
**

**Supplemental Figure 1.** The dynamic changes of laboratory indexes within 30 days in patients with liver disease, using the generalized additive mixed model. (A) TBIL, (B) DIBL, (C) IBIL, (D) Cre, (E) INR, (F) WBC. TBIL, total bilirubin; DBIL, direct bilirubin; IBIL, indirect bilirubin; Cre, creatinine; INR, international normalized ratio; WBC, white blood cells.

**Supplemental Table 1.** Relationship between changes in laboratory indexes and NRBCs in patients with liver disease within 30 days.

| **Indexes** | **NRBCs** | **Day** | **Day × NRBCs** |
| --- | --- | --- | --- |
| **TBIL (**mg/dL**)** | -0.26 (-1.47, 0.95) | -0.076 (-0.09, -0.06) * | 0.16 (0.13, 0.19) * |
| **DBIL** (mg/dL) | -1.48 (-3.04, 0.08) | 0.10 (0.03, 0.16) * | 0.26 (0.15, 0.36) * |
| **IBIL** (mg/dL) | -0.46 (-1.08, 0.15) | 0.02 (-0.01, 0.05) | 0.05 (0.01, 0.10) * |
| **Cre (**mg/dL**)** | 0.03 (-0.18, 0.24) | -0.01 (-0.01, -0.01) * | 0.01 (0.004, 0.01) * |
| **INR** | 0.001 (-0.12, 0.12) | -0.01 (-0.02, -0.01) * | 0.01 (0.004, 0.01) * |
| **WBC (**K/uL**)** | 0.44 (-0.98, 1.85) | 0.01 (-0.02, 0.04) | 0.09 (0.05, 0.13) * |

Results are expressed as β (95% CI). *P <0.05. CI, confidence interval; TBIL, total bilirubin; DBIL, direct bilirubin; IBIL, indirect bilirubin; Cre, creatinine; INR, international normalized ratio; WBC, white blood cells. NRBCs indicated the difference of indexes at day 0 in high NRBCs group compared with low NRBCs group. Day indicated the mean of the increasing of indexes daily in low NRBCs group. Day × NRBCs indicated the average increasing of indexes daily in high NRBCs group compared with low NRBCs group.
